# A patient with Marfan’s syndrome who developed an acute aortic dissection at 28 weeks of pregnancy treated with aortic root replacement

**DOI:** 10.1007/s11748-021-01596-3

**Published:** 2021-02-07

**Authors:** Riki Sumiyoshi, Hideki Morita, Kento Fujii, Takehiro Shirasughi, Hiroyuki Kawaura, Masakazu Aoki, Hiroshi Nagano

**Affiliations:** Department of Cardiovascular Surgery, Saitama Red Cross Hospital, 1-5 Shintoshin, Chuo-ku, Saitama-shi, Saitama 330-8553 Japan

**Keywords:** Marfan’s syndrome, Pregnancy, Acute aortic dissection, Aortic root surgery

## Abstract

The patient was a 31-year-old pregnant woman who gave birth to her first child by vaginal delivery 7 years ago. She was diagnosed with Marfan’s syndrome based on physical findings; however, the condition was not diagnosed before the onset. The patient developed acute aortic dissection at 28 weeks of pregnancy. A cesarean section was first performed to save the patient’s life; then, a total hysterectomy was performed to prevent the risk of postpartum hemorrhage. Furthermore, aortic root replacement was performed using a temporary mechanical valve. The patient and her child have survived without any complications.

## Introduction

Pregnancy increases the circulating blood volume in the body by 1.5 times compared with during non-pregnant state, which increases cardiac output and heart rate [1]. In addition, when aortic tunica media elastic fibers become fragile due to an increase in hormones during pregnancy [2, 3], aortic dilatation occurs and resistance decreases. These are fit-for-purpose changes for normal pregnant women; however, in patients with connective tissue diseases, such as Marfan’s syndrome, medical conditions may worsen, and if aortic dissection develops during pregnancy, both the mother and child are at high risks.

We performed aortic root replacement on a patient with Marfan’s syndrome who developed acute type A aortic dissection at 28 weeks of pregnancy, observed a good course, and hereby reported the case.

## Case report

The patient was a 31-year-old woman in the 28th week of gestation who had delivered her first child vaginally 7 years earlier. During pregnancy, there were no abnormalities in either the mother or infant. The patient presented at her regular obstetrics and gynecology department complaining of sudden left chest pain. As the infant was confirmed to be healthy, the patient was referred to her regular physician. A diagnosis of acute type A aortic dissection was made based on echocardiography and the patient was transferred to our institution by ambulance to undergo surgery.

The patient’s medical history was unremarkable. Her family history revealed that her biological father had experienced aortic dissection. The patient was 173 cm tall, weighed 61 kg, and was lucid upon arrival at our institution. She had long hands and feet, arachnodactyly of the fingers and toes, limited development of the elbow joints, skin striae, and pectus carinatum. Auscultation revealed a to-and-fro murmur. The patient’s blood pressure was 147/80 mmHg, and her heart rate was 80 beats/min.

Electrocardiography showed a sinus rhythm and no ST changes. A chest X-ray revealed a cardiothoracic ratio of 53% and enlargement of the left first arch. An ultrasound cardiogram revealed an enlarged aortic root (65 mm) and a flap and false lumen just above the right coronary cusp. The aortic valve was tricuspid, and severe aortic valve regulation was observed.

**Fig. 1:**
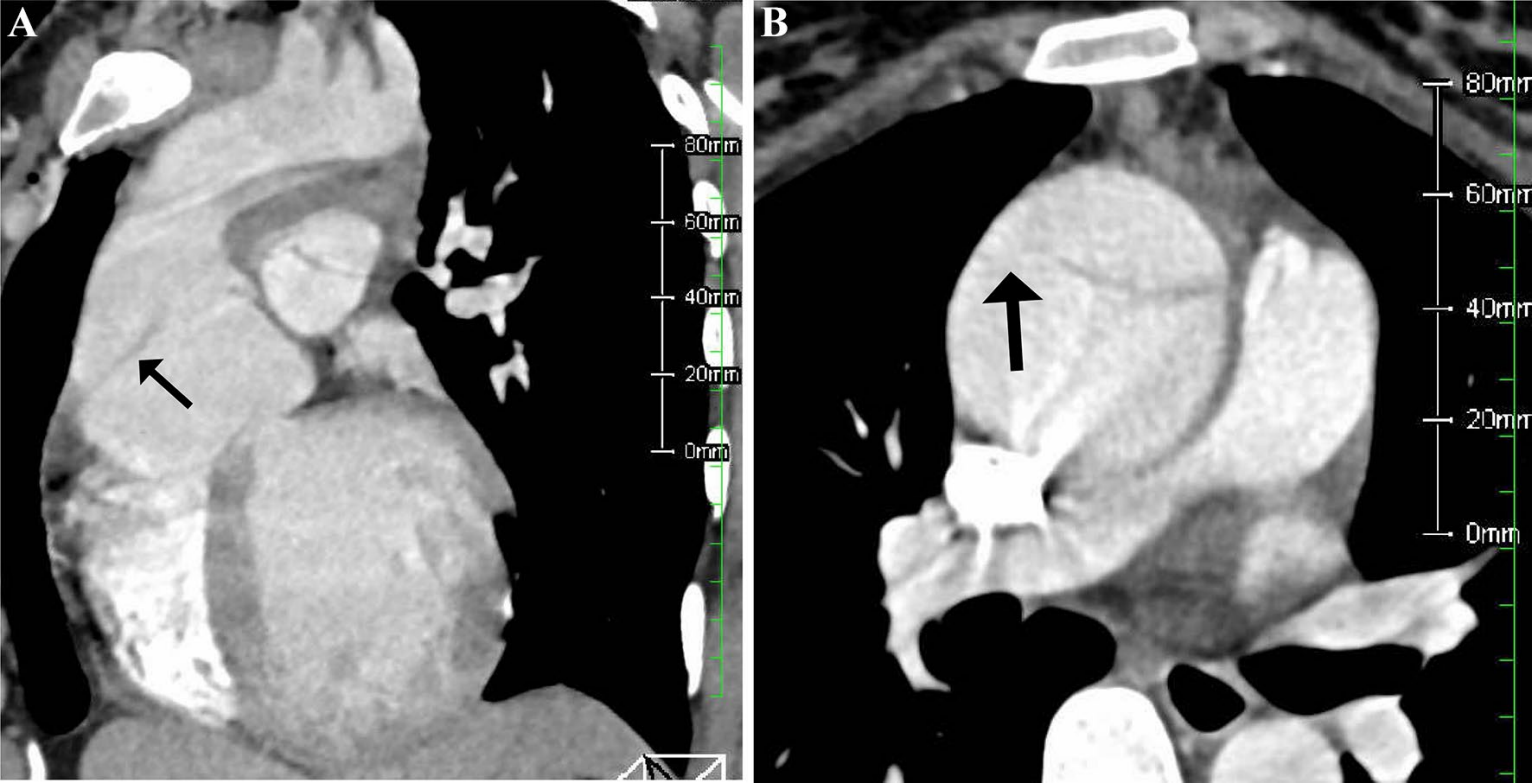
**a** Dilation and dissection of the aortic root. **b** Initial flap observed in the ascending aorta

**Fig. 2:**
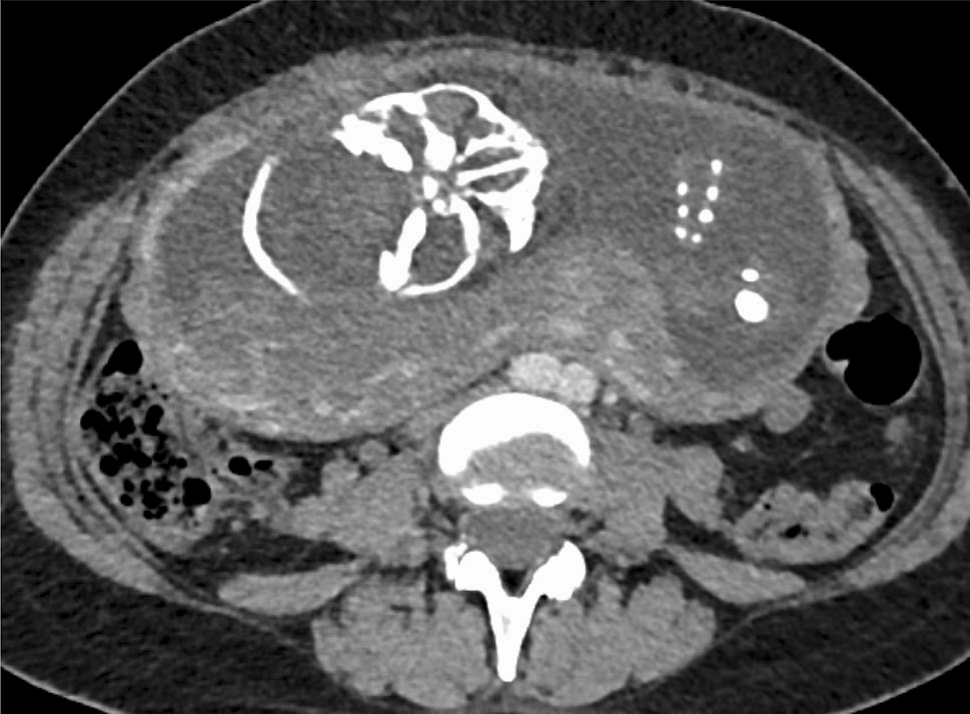
Compression of the interior vena cava by the uterus and fetus

Contrast-enhanced chest and abdomen computed tomography showed a 65-mm-diameter ascending aorta and a 69-mm-diameter sinus of Valsalva. There was an initial flap limited to the ascending aorta and the false lumen was patent (Fig. 1a, b). No dissection was observed beyond the aortic arch. Pericardial effusion was not observed. The fetus was confirmed in the abdominal cavity, and blood vessels were compressed (Fig. 2).

DeBakey type 2 aortic dissection was diagnosed. After consultation with the obstetrics and gynecology department at our institution, a decision was made to perform cardiac aortic surgery after cesarean section and hysterectomy.

Disinfection and draping, as well as an extracorporeal circuit, were set up to allow for open chest surgery to be performed immediately if the patient’s hemodynamics became unstable during the cesarean section or hysterectomy. The cesarean section and total hysterectomy were performed first. The infant weighed 1173 g with an Apgar score of 2/10 at 5 min. The infant was immediately transferred to the neonatal intensive-care unit. Aortic surgery was performed after abdominal closure.

The aortic root was dilated in a pyriform manner. Blood was delivered to the ascending aorta in the non-dissected area by the Seldinger method, and extracorporeal circulation was established with blood removal from the right atrium via two tubes. A left ventricular vent was inserted, and the ascending aorta was occluded when ventricular fibrillation occurred. The ascending aorta was dissected, and cardiac arrest was caused by selective antegrade injection of cardioplegic solution. The central temperature was cooled to 20 °C and circulatory arrest was induced. The aorta was transected at the center of the brachiocephalic trunk, a prosthetic graft (26 mm Triplex™; Terumo Corporation, Tokyo, Japan) was anastomosed, and extracorporeal circulation was resumed.

**Fig. 3:**
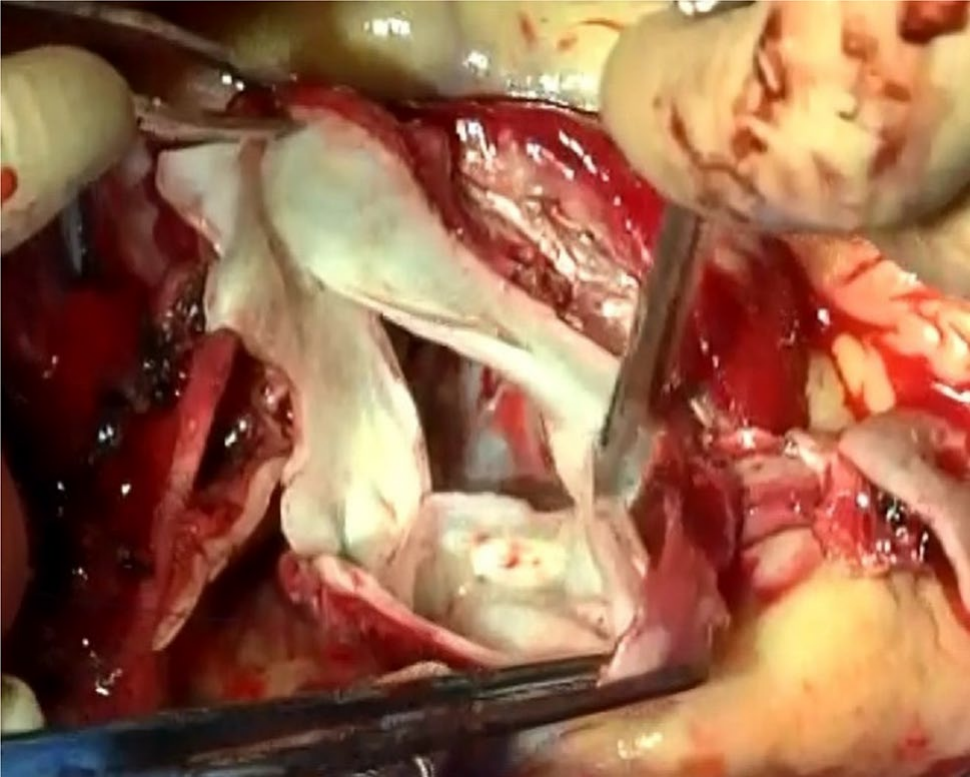
A thin and fragile aortic valve

The aortic valve was tricuspid, and the sinotubular junction and sinus of Valsalva were markedly enlarged. The valve cusp became thin and fragile (Fig. 3); thus, root replacement was performed. An artificial valve (25 mm SJM Regent; Abbott, Abbott Park, IL) and a prosthetic graft (30 mm Gelweave Valsalva; Vascutek Terumo Inc., Scotland, UK) were sutured to the aortic annulus. Both the left and right coronary arteries were reconstructed using the Carrel patch technique.

The duration of the cesarean section and total hysterectomy was 1 h and 30 min, and the time to transfer to aortic surgery was 10 min. The duration of aortic surgery was 5 h and 58 min. The extracorporeal circulation time was 3 h and 28 min, the aortic cross-clamp time was 2 h and 48 min, and the circulatory arrest time was 33 min. A blood transfusion was performed with two units of red blood cells, two units of fresh frozen plasma, and 25 units of platelet concentrates.

Postoperatively, the orotracheal intubation was extubated the day after the surgery. The patient was transferred to the general ward from the coronary care unit on day 7 after surgery. The patient had an uneventful postoperative course and was discharged on day 18. The patient is currently being treated as an outpatient. The infant was discharged on day 86 after delivery without any disabilities.

## Discussion

The incidence of aortic dissection in women younger than 40 years of age is 0.4 in 100,000, and approximately half of these cases are said to be related to pregnancy [4]. The mortality rate is high, with 21.4% of patients dying before being seen at a hospital, and mortality rates of 60.7% and 75% on the first and second days after onset, respectively [5]. Between 2010 and 2017, 33 maternal deaths (376 cases) in Japan were attributed to cardiovascular system disorders, with acute aortic dissection accounting for approximately half of the deaths (15 cases) [6]. In some reports, cesarean section and aortic surgery were performed in two stages to reduce the risk of hemorrhage. If acute aortic dissection occurs, the mortality rate within 2 days after onset is so high that performing aortic surgery later is very risky. Thus, it is preferable to perform surgery for aortic dissection as soon as possible after cesarean section, unless the patient can safely wait for surgery.

During pregnancy, approximately 12% of the cardiac output flows through the uterus and placenta [7, 8], and childbirth can remove the compression on large vessels and increase the cardiac output by 60–80% [9]. The uterus in pregnant women is generally thought to place pressure on the large blood vessels, thereby adversely affecting maternal circulation at 20 weeks of gestation or later [10]. At 28 weeks of gestation, the mean fetal weight is 1100 g, the mean placental weight is 300–400 g [11], and the mean amniotic fluid is approximately 800 g. Aortic surgery is typically performed first if the patient is hemodynamically unstable. However, during hypothermic extracorporeal circulation, the presence of an infant compressing the large blood vessels and requiring a large volume of circulating blood could compromise the survival of the mother. The fetal demise rate under an artificial heart–lung machine is 20% [12]; however, hypothermic circulatory arrest has been reported to cause increased fetal demise and severe brain complications [12, 13]. In addition, the neonatal survival rate in Japan is > 80% after 24 weeks [14]. In the case of this patient, the hemodynamics was steady and there was time to perform cesarean section first; thus, the fetus was delivered by cesarean section with maternal lifesaving as the priority.

Postpartum hemorrhage occurs in 2–11% of all deliveries, and 75% of cases of abnormal bleeding within the first 24 h postpartum have been shown to be caused by atonic bleeding [15]. Treatments such as blood transfusion, oxytocin administration, intrauterine balloons, and uterine bimanual compression are usually used. However, if this occurs during open-heart surgery, it can be fatal as manual treatment cannot be performed and heparin is being administered, thereby promoting bleeding. Thus, we decided that the safest option would be to perform total hysterectomy to prevent atonic bleeding. In this case, the baby was the second child and there was a good chance for the baby to be saved after delivery; therefore, the patient did not wish for future pregnancies and agreed to undergo hysterectomy. Even if the patient hoped for future pregnancies, as it is extremely difficult to save lives if atonic hemorrhage occurs during an open-heart surgery, it was safer to gain the family’s understanding and perform a total hysterectomy for saving the mother’s life.

## Conclusion

We performed a cesarean section, a total hysterectomy, and an aortic root replacement in one go on a pregnant patient with Marfan’s syndrome who developed acute aortic dissection at 28 weeks of pregnancy. Both the mother and child were saved and had a good course.
